# Respiratory Insufficiency Correlated Strongly with Mortality of Rodents Infected with West Nile Virus

**DOI:** 10.1371/journal.pone.0038672

**Published:** 2012-06-14

**Authors:** John D. Morrey, Venkatraman Siddharthan, Hong Wang, Jeffery O. Hall

**Affiliations:** Institute for Antiviral Research, Department of Animal, Dairy, and Veterinary Sciences, Utah State University, Logan, Utah, United States of America; University of Texas Medical Branch, United States of America

## Abstract

West Nile virus (WNV) disease can be fatal for high-risk patients. Since WNV or its antigens have been identified in multiple anatomical locations of the central nervous system of persons or rodent models, one cannot know where to investigate the actual mechanism of mortality without careful studies in animal models. In this study, depressed respiratory functions measured by plethysmography correlated strongly with mortality. This respiratory distress, as well as reduced oxygen saturation, occurred beginning as early as 4 days before mortality. Affected medullary respiratory control cells may have contributed to the animals' respiratory insufficiency, because WNV antigen staining was present in neurons located in the ventrolateral medulla. Starvation or dehydration would be irrelevant in people, but could cause death in rodents due to lethargy or loss of appetite. Animal experiments were performed to exclude this possibility. Plasma ketones were increased in moribund infected hamsters, but late-stage starvation markers were not apparent. Moreover, daily subcutaneous administration of 5% dextrose in physiological saline solution did not improve survival or other disease signs. Therefore, infected hamsters did not die from starvation or dehydration. No cerebral edema was apparent in WNV- or sham-infected hamsters as determined by comparing wet-to-total weight ratios of brains, or by evaluating blood-brain-barrier permeability using Evans blue dye penetration into brains. Limited vasculitis was present in the right atrium of the heart of infected hamsters, but abnormal electrocardiograms for several days leading up to mortality did not occur. Since respiratory insufficiency was strongly correlated with mortality more than any other pathological parameter, it is the likely cause of death in rodents. These animal data and a poor prognosis for persons with respiratory insufficiency support the hypothesis that neurological lesions affecting respiratory function may be the primary cause of human WNV-induced death.

## Introduction

West Nile virus infection is fatal in less than 1% of human cases [1]. The fatality rate in experimentally infected mice and hamsters is typically reported to be between 20–80% [2], [3], [4]; therefore, rodents provide an opportunity to investigate the cause of WNV death that might be applicable to human fatality. In human and rodent infections, the cause of death appears to be neurological, but definitive studies have not been performed. Knowing the cause of death is necessary to identify the anatomical location, type, and cause of the responsible lesions, which is ultimately important in the management and treatment of West Nile neurological disease (WNND).

Careful examination of the human signs and symptoms can provide clues as to WNV pathogenesis and cause of death. The WNV fever stage includes flu-like symptoms such as fever, headaches, chills, swollen lymph nodes, and excessive sweating. Some symptoms may reflect alterations in the autonomic nervous system, such as gastrointestinal symptoms, fatigue [5], or more rarely reported cardiac, bladder, or urinary dysfunctions [6], [7]. WNV encephalitis stage includes symptoms such as acute flaccid paralysis, limb weakness, and poliomyelitis-like syndrome, which probably reflect infection in motor neurons of the anterior horn of the spinal cord. Severe autonomic dysfunctions may contribute to respiratory insufficiency and cardiac arrhythmias. Respiratory insufficiency is recognized to have a poor-prognosis in human with West Nile neurological disease WNND [8]. Neurological sequelae of motor dysfunctions are well documented, particularly for limb weakness and paralysis [9], [10]. Patients have reported persistent fatigue, memory problems, and word-finding difficulty suggestive of abnormalities in attention and executive functions [11]. In a cohort of 54 patients approximately 1.5 years after WNV diagnosis, a minority of subjects had persistent neurocognitive deficits. These clinical findings suggest involvement of areas of the CNS controlling motor functions and possibly in areas controlling higher-order central nervous system (CNS) functions.

Experimental approaches have revealed that the WNND in rodent models is not inconsistent with the human disease; therefore, rodent models can be used to investigate the mechanism of death, as it may be relevant in fatal human cases. WNV infects motor neurons in the spinal cord of rodents and people to cause poliomyelitis-like infection and occasional paralysis [2], [12]. Infection in the hamster model causes histopathology in the lumbosacral and cervical cord, and to a lesser extent in the thoracic cord [13], [14]. Loss of motor units from infection of the lumbosacral cord in hamsters correlates with hind limb paralysis [13]. Involvement of spinal cord implies that it could also affect sympathetic autonomic functions of respiratory, cardiac, and gastrointestinal tract functions [15].

WNV infection and associated histopathology is extensive in the brainstem of rodents [16], [17] and humans [18], [19], [20]. The brainstem not only contains nerve tracts connecting the cerebrum and cerebellum with the spinal cord, but also possesses integrative functions some of which are necessary for life, i.e., cardiovascular system and respiratory control. The importance of WNV infection of the brainstem is illustrated in the hamster model by the association of suppressed diaphragmatic electromyographs (EMGs) with infection of the medulla oblongata [17]. WNV-induced lesions in the limbic system may be associated with measurable loss of spatial memory in hamsters [21], which suggests that involvement of higher-order functions may be pertinent in rodent models [13]. In short, the human and rodent model data indicate that the mechanism of death may be due to loss of motor neurons, autonomic nervous system dysfunction, loss of cardiorespiratory function from infection of the spinal cord, brainstem, or perhaps areas of the brain controlling higher order functions. The cause of death of WNV infection in rodents was investigated in this study; wherein, the main contribution is that respiratory insufficiency was identified to be highly associated with mortality, unlike all other parameters evaluated.

## Results

**Figure 1:**
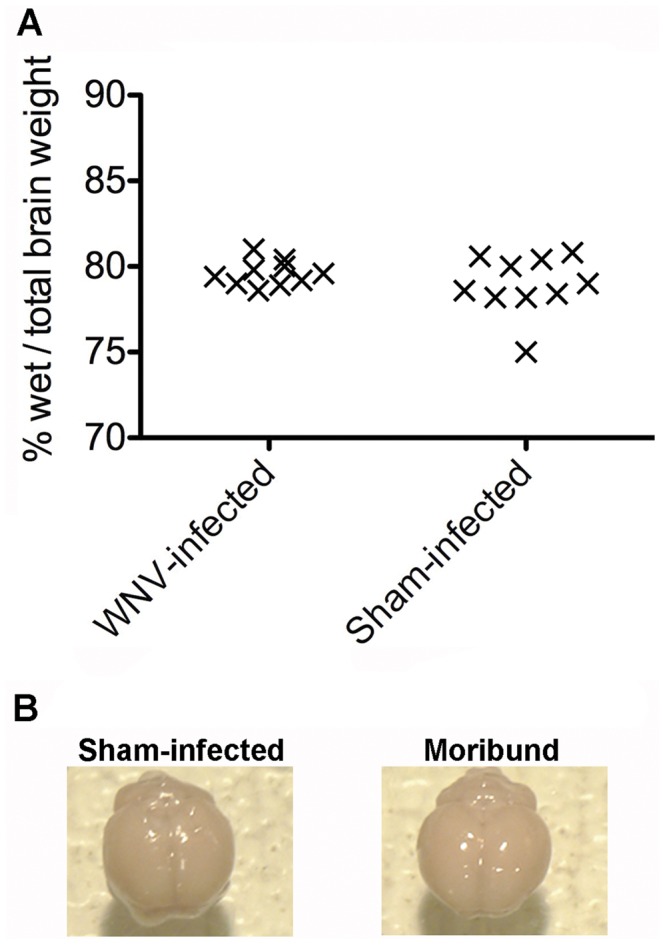
Lack of evidence for brain edema in WNV-infected moribund hamsters. A) Hamsters were injected s.c. with WNV or sham. Brains were collected from WNV-infected hamsters when they were moribund. A corresponding sham-injected hamster was necropsied with every WNV-moribund hamster. Weights of brains before and after drying at 110°C for 24 hr were used to calculate the ratio of wet/total weight of each brain as an indication of edema. B) Fifteen hamsters were injected with WNV and five were injected with sham. When one WNV-injected hamster became moribund, a corresponding non-moribund WNV-injected hamster was selected for i.v. injection of Evans blue dye. Four hours later, brains were examined for any blue color due to breakdown of the blood-brain-barrier. Examples of brains from WNV- and sham-injected hamsters are shown. No blue color was evident in all of the WNV- or sham-injected hamsters.

Different disease states were evaluated as possible causes of death in WNV-infected rodents, i.e., cerebral edema, starvation, seizures, and vital organ failure due to disease of the organ or autonomic nervous system failure. Brain edema was not the cause of death since there was no difference in the ratio of wet/total brain weight between moribund WNV-infected and sham-infected hamsters (Figure 1A). Moreover, no difference in the Evans blue dye uptake was observed when comparing the brains of WNV-infected moribund hamsters and sham-infected hamsters as a measure of blood-brain-barrier permeability (Figure 1B).

**Figure 2:**
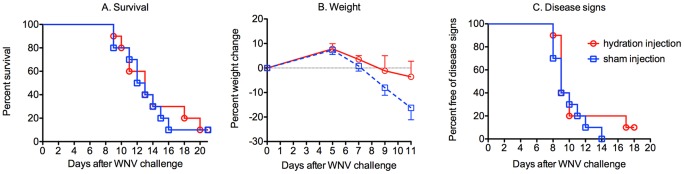
Hydration supportive therapy for WNV-infected hamsters. Six days after viral challenge just before the earliest disease signs appeared and up to 14 days after viral challenge, 10 hamsters were treated s.c. with D5 dextrose solution (5% dextrose, 0.9% physiological saline); and 10 hamsters were sham-injected. The animals were monitored through day 21 for A) mortality, B) weight change, and C) disease signs such as hind limb paralysis, front leg tremors, eye lacrimation, and diarrhea. Mortality was considered a disease sign too when constructing the graph. ***P≤0.001 using log rank test.

Since rodents used in infectious disease models [22] typically lose considerable amounts of body weight, including those infected with WNV [23], [24], we investigated whether rodents succumb to the disease simply because of dehydration or starvation from a loss of appetite or supportive care. Six days after viral challenge just before the earliest disease signs appeared and up to 14 days after viral challenge, 10 hamsters were treated intraperitoneally (i.p.) with 10 mL/day of D5 dextrose solution (5% dextrose, 0.9% physiological saline); and 10 hamsters were sham-injected without administration of any fluid. The animals were monitored through day 21 for mortality, weight change, and disease signs such as hind limb paralysis, front limb tremors, eye lacrimation, and diarrhea. Hydration did not improve survival or disease signs, so animals were not dying from dehydration (Figure 2A and 2C).

**Figure 3:**
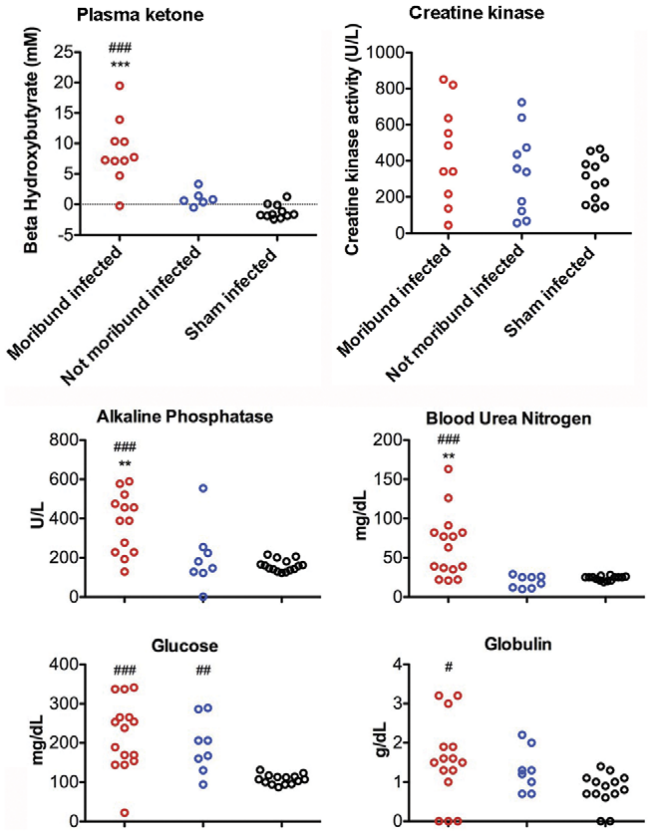
Plasma ketone, creatine kinase, and blood chemistries in WNV-infected moribund hamsters. Twenty-three hamsters were injected with WNV and fifteen were injected with sham. When one WNV-injected hamster became moribund, the plasma was obtained from a corresponding non-moribund WNV-injected hamster. Numbers were unequal between the assays, because of insufficient sample volume to perform all assays. ^###^P≤0.001 compared to sham-infected, ***P≤0.001 compared to not-moribund infected using t-test.

To determine if clinical tests could provide information as to the cause of death, such as starvation, a cohort of WNV-infected hamsters were observed for near-death condition (moribund). When one WNV-injected hamster became moribund, blood and tissues were collected from that hamster and from a corresponding not-moribund WNV-injected hamster. Sham-infected hamsters were also included. Plasma ketones, alkaline phosphatase (ALP), blood urea nitrogen (BUN), and globulin concentrations were statistically increased in moribund hamsters as compared to not-moribund or sham-infected hamsters (Figure 3). Glucose (GLU) was statistically elevated in both the moribund and not-moribund infected hamsters as compared to the sham-infected hamsters. All other chemistry panel tests were unaffected (alanine aminotransferase, albumin, creatinine, sodium, potassium, calcium, phosphorous, amylase, and total protein).

In agreement with previous observations [25], vasculitis was observed within the right atrium of the heart of infected hamsters, along with moderate aggregates of lymphocytes within the myocardium of the interventricular septum (Table 1). Nevertheless, these histopathological effects did not cause a significant increase of creatine kinase as a marker for muscle tissue damage, including the heart (Figure 3). Kidney, intestinal, or liver histology was unremarkable and would not account for any mortality (Table 1).

**Table 1:** H&E histological analysis of tissues from WNV-infected hamsters.

Animal Number	Days to Necropsy	Condition	Heart	Liver	Stomach	Kidney
109	9	Not-moribund	A	C	NS	NS
125	9	Not-moribund	A	C	NS	NS
114	11	Not-moribund	F	NS	NS	NS
106	12	Not-moribund	A	NS	D	NS
122	13	Not-moribund	NS	NS	NS	NS
101	14	Not-moribund	A	NS	NS	NS
107	14	Not-moribund	E	NS	NS	NS
103	19	Not-moribund	NS	B	NS	NS
113	9	Moribund	A	NS	NS	NS
115	9	Moribund	A	NS	NS	NS
108	11	Moribund	A	NS	NS	NS
117	11	Moribund	A	NS	NS	NS
118	11	Moribund	A	NS	NS	NS
105	13	Moribund	A	NS	D	NS
120	13	Moribund	A	NS	D, G	NS
110	14	Moribund	NS	NS	NS	NS
111	14	Moribund	A	C	D	NS
112	19	Moribund	NS	C	NS	NS
119	19	Moribund	A	NS	NS	NS
123	19	Moribund	NS	B	D	NS
124	14	Moribund	A	NS	NS	NS

NS: No significant histological lesions.

A: Mild focal acute degeneration of the tunica media of a large vessel adjacent to the right atrium. Mixed cell marginalization along the intima of this vessel. Moderate numbers of lymphocytes and plasma cells infiltrate connective tissue bordering the affected vessel wall.

B: Hydropic degeneration of hepatocytes.

C: Scattered small groups of hepatocytes containing single to multiple, small intracytoplasmic lipid vacuoles.

D: Moderate numbers of mixed mononuclear cells including a population of eosinophils and histiocytes containing dull basophilic intracytoplasmic material.

E: Localized neutrophilic infiltration of the tissue anchoring the valvular leaflets on the left side.

F: Moderate aggregates of lymphocytes and fewer plasma cellular infiltrate the myocardium of the interventricular septum (cranial) and the tip of the right ventricular wall (approximately the AV junction).

G: In colon, segmental moderate infiltration of the muscularis mucosa by bands of neutrophils.

**Figure 4:**
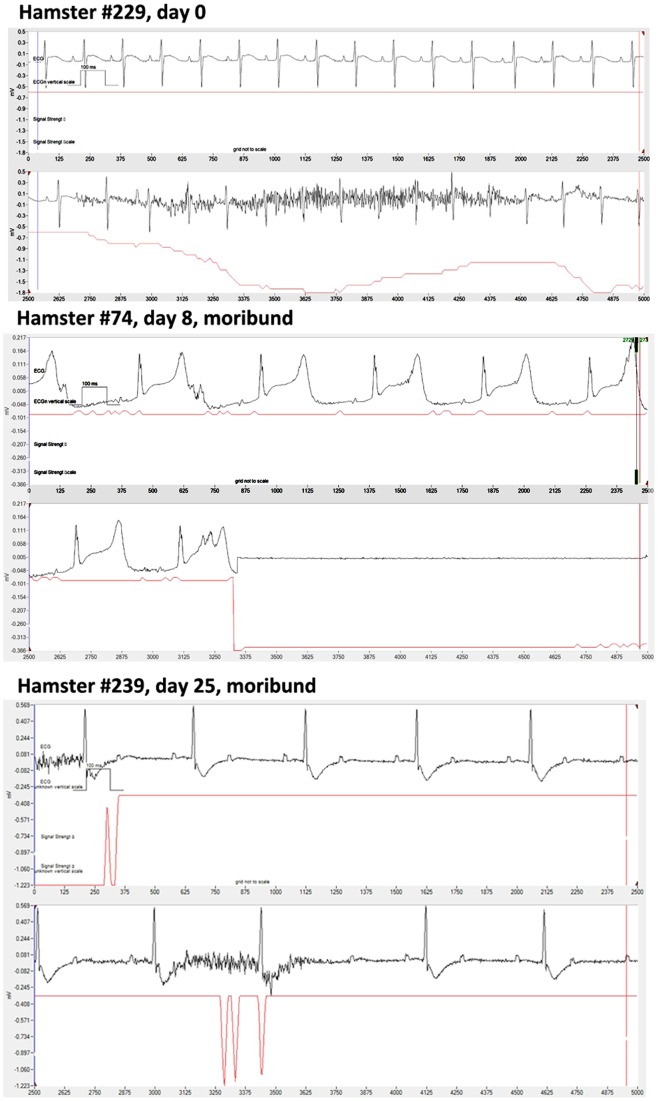
Telemetric 2-lead electrocardiograms from WNV-infected hamsters taken over the course of disease. Example of normal ECG from a hamster (#229) at day 0 where the erroneous signal was identified by a drop in the signal strength. Example of abnormal ECGs of two infected hamsters at time of death (#74) and within 6 hr of death (#239).

To investigate heart disease as a possible cause of death, ECGs from resting hamsters were monitored by telemetry over the course of disease (Figure 4). To eliminate erroneous ECG tracings using the computer software, poor tracings were identified and eliminated when the signal strength was low. For example, hamster #229 had normal ECG where the erroneous signals were identified by a drop in signal strength. Within the limits of a 2-lead telemetry, ECG functions of the computer program allowed us to determine that there were no consistent pathological patterns (ST elevation, ectopic P waves, arrhythmia, tachycardia or bradycardia, and fibrillation) to account for heart disease as the cause of death. We did observe varied abnormal ECGs occurring within hours of the death (Figure 4) that were consistent with the death process, but we did not interpret these pathological events as the cause of mortality developing from the WNND over the course of days. Moreover, the ECG abnormalities just before mortality varied between animals.

**Figure 5:**
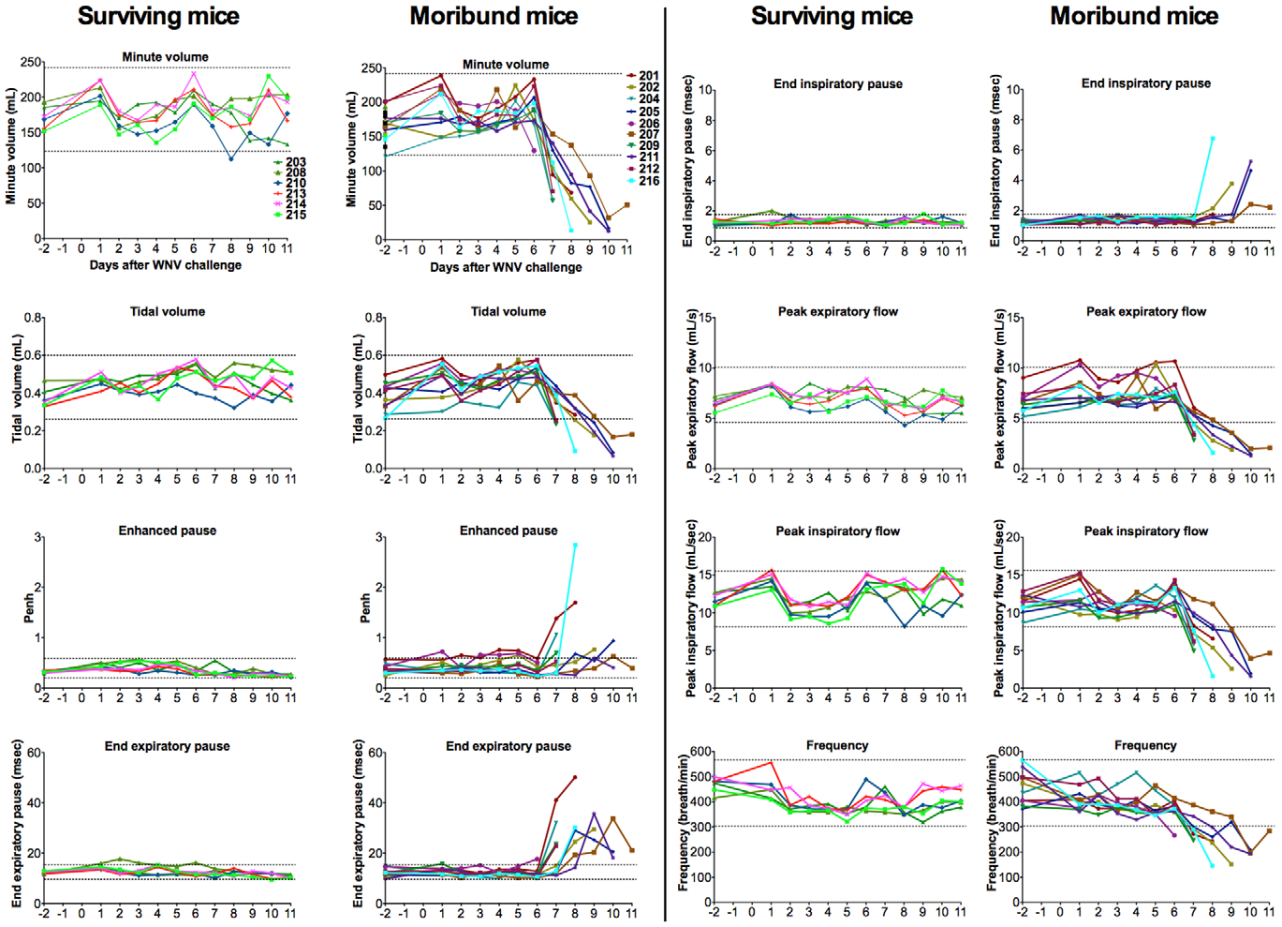
Plethysmography of C57BL/7 mice infected with WNV. Sixteen mice were injected s.c. with WNV. Plethysmograph readings were obtained 2 days before viral challenge and from days 1 to 11 after viral challenge. Data were divided between the surviving or moribund mice. The dotted lines are two standard deviations of the mean of readings on days −2, 1, and 2 for all animals.

Since we have previously reported that WNV infection causes suppression of electromyography of the diaphragm [17] and autonomic nervous system dysfunction [25], respiratory functions were evaluated in 16 WNV-infected C57BL/6 mice using whole body plethysmography (Figure 5). Ten of the mice became moribund within an 11-day period. Six of the mice survived through 21 days. Eight different quantifiable parameters were calculated from the plethysmograph tracings using commercially available software. The trends of the parameters correlated remarkably well with the survival or death of mice. Two standard deviations of the mean (95% confidence) of data at days −2, 1, and 2 for all animals were calculated and represented as dotted lines in Figure 5. Data within this range were considered normal. Nearly all data points for moribund animals were outside of normal readings by the time that they succumbed to the disease for minute volume, tidal volume, end expiratory pause, peak expiratory flow, peak inspiratory flow, and frequency. Enhanced pause (Penh), and end inspiratory pause reflected the same pattern, but some of the values at time of mortality were within normal value range. The data of the animal succumbing to disease at the earliest time point at day 6 (#206) were trending to abnormality, but were within normal values with all the respiratory parameters shown except for end expiratory pause and frequency. These data tend to support reduced diaphragmatic EMG data [17], which could be caused by loss of neurological respiratory motor function or respiratory drive [26].

**Figure 6:**
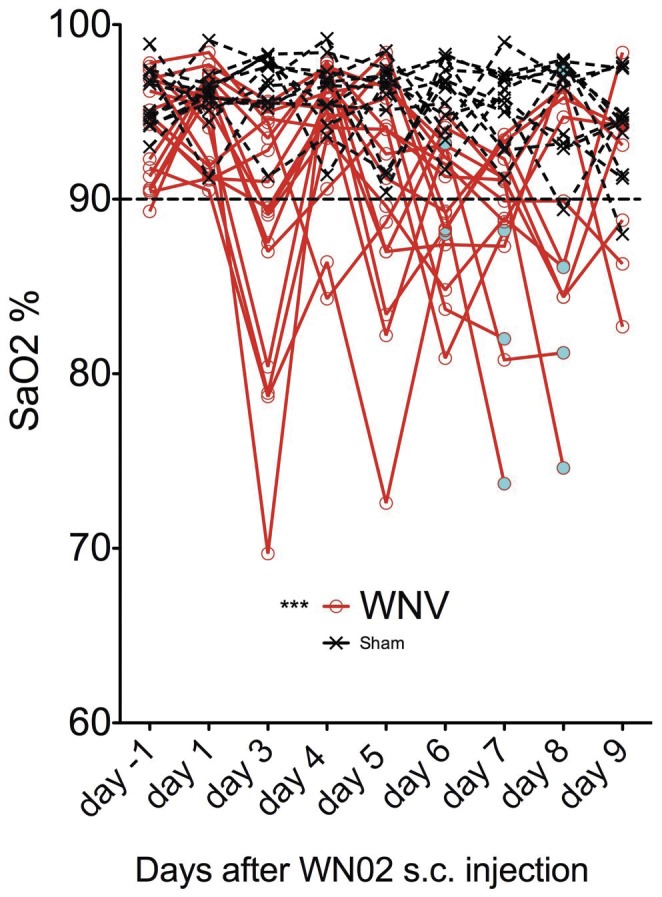
Pulse oximetry of BALB/c mice infected with WNV or sham. White female BALB/c mice (>7 weeks old) were infected s.c. with WNV and monitored over time for Sa0_2_ using pulse oximetry. Seventeen animals were included in each group. The cyan-colored circles identified animals that had become moribund. ***P≤0.001 using one-way analysis of variance with Newman-Keuls comparison.

To measure the presence of respiratory insufficiency, the oxygen saturation (SaO_2_) was measured in alert BALB/c mice with rodent pulse oximetry [27] (Figure 6). The cyan-colored circles identified animals that had become moribund. Seven out of nine animals that succumbed to the disease had reduced SaO_2_ below 90%. The other two moribund animals had values of 93.2% and 97.4%. Even though the readings varied from day to day within each mouse, and the correlation with mortality and low SaO_2_ was not perfect, the overall readings indicated that mice infected with WNV had SaO_2_ values below the sham-injected mice. A one-way analysis of variance revealed a statistically significant difference (P≤0.001) between the two groups. For practical application, pulse oximetry was more variable presumably because of inaccuracy, and the plethysmography test was much easier to apply than pulse oximetry in mice. The plethysmography yielded more consistent results, and provided the better functional assay for respiratory distress in mice. Oxygen saturation was very low (<80%) at day 2, whereas, the plethysmography declined no sooner than day 6. The reasons for this was not investigated, but BALB/c mice were used in the pulse oximetry experiment and C57BL/6 mice were used in the plethysmography experiment. These two strains do behave differently to WNV infection [28].

**Figure 7:**
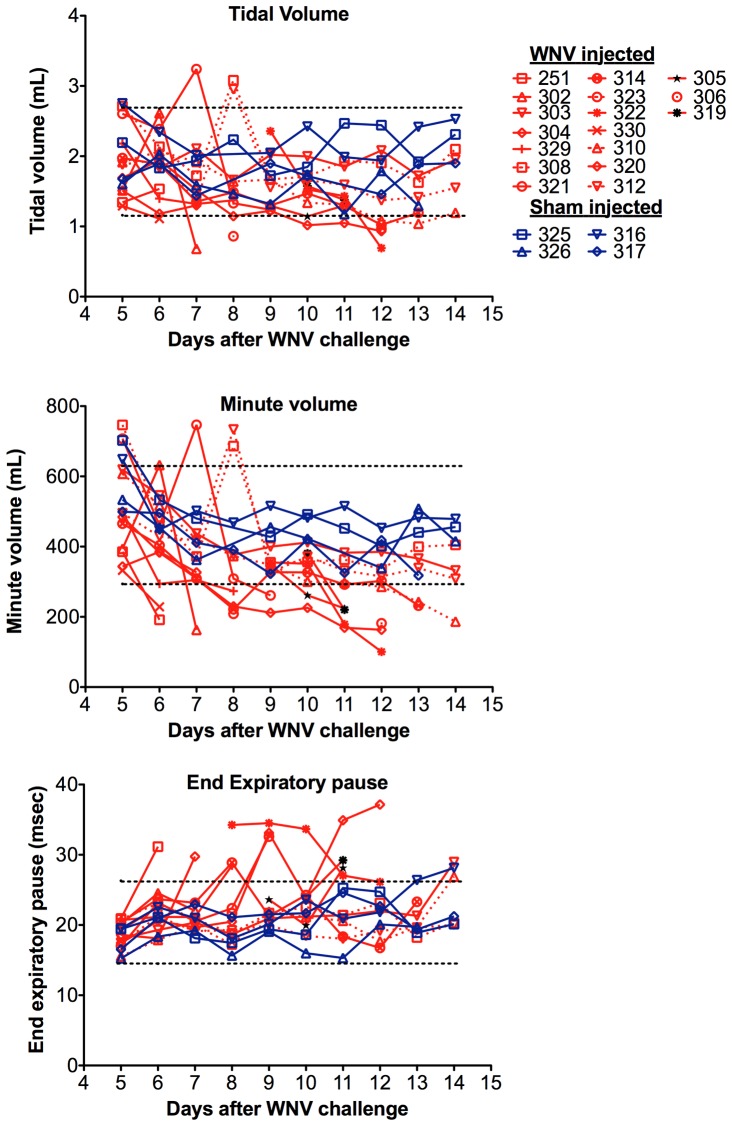
Respiratory function as measured by plethysmography of hamsters infected with WNV. All but three animals (#251, #303, #304) eventually became moribund. The dotted lines are two standard deviations of the means (n = 46) of sham-infected animals.

Plethysmography was performed with WNV- and sham-injected hamsters. When infected hamsters became moribund, as identified by a line terminated by a symbol before day 14 in Figure 7, their respiratory functions were consistently lower than the readings of earlier time points, and generally lower than other surviving hamsters. No moribund animals had elevated respiratory functions. Three infected hamsters (#303, 308, 312) survived to day 14 and had the higher minute volume values as compared to #310 that became moribund at day 14. The minute volumes of 11/14 animals were outside two standard deviations of the sham-infected means. The end expiratory volumes of 9/14 moribund animals were outside two standard deviations. The tidal volumes of only 5/14 animals were outside the two standard deviations. Animals with respiratory values below two standard deviations of sham-infected animals were predictive to die from WNV disease. Minute volume was the best parameter in identifying animals that were moribund, followed by end expiratory pause, and other parameters such as tidal volume. The hamster plethysmography readings were not nearly as clear as the mouse readings, which was due to the observation that the mouse plethymographic tracings were more regular, whereas the hamster tracings contained far more sniffing patterns and other irregular patterns (data not shown). Due to more consistent breathing patterns with the mice as compared to the hamsters, the mouse model was better for determining the effects of WNV infection on respiratory functions.

**Figure 8:**
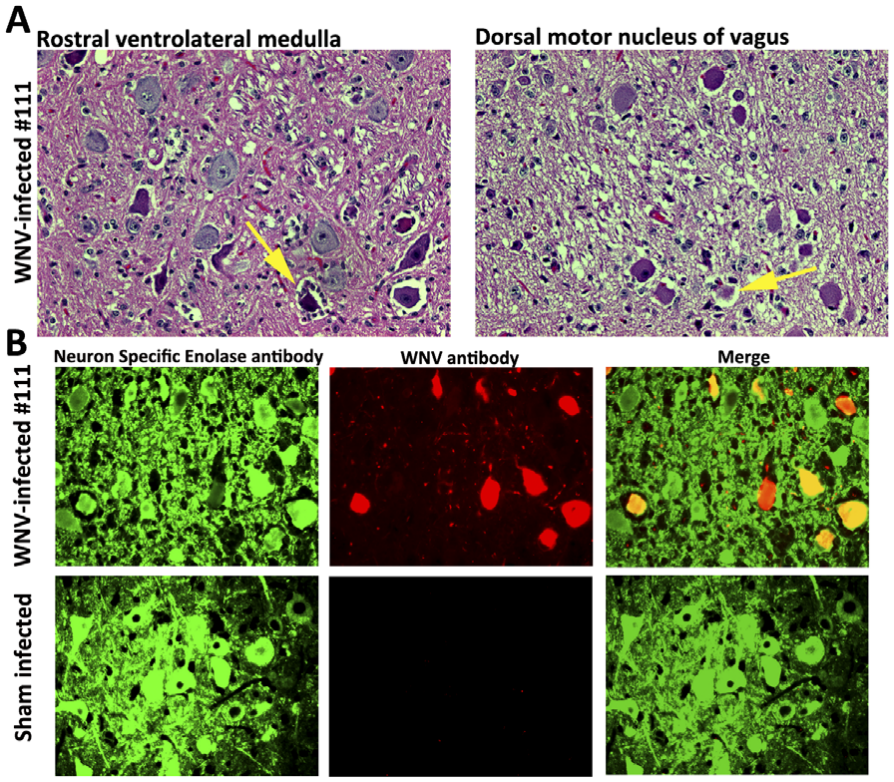
Histopathology of moribund WNV-infected hamster (#111) with respiratory distress. A) H&E staining of rostral ventrolateral medulla containing respiratory control cells and dorsal motor nucleus of vagus. Yellow arrows indicate necrotic neurons. B) Immunohistochemical staining for neuron specific enolase (green) and WNV (red).

The brainstem from a WNV-infected hamster (#111) with a minute volume well below 2 standard deviations of the mean was examined for histopathology. (Figure 8A). The rostral ventrolateral medulla, containing respiratory control neurons [29], was heavily affected with gliosis, acute degenerative neuronal necrosis, edema around neurons, and perivascular cuffing. The dorsal motor nucleus vagus contained early pre-acute changes such as early neuronal necrosis and increased gliosis. Moreover, most of the immunofluorescent staining for WNV envelope was co-localized with neuron specific enolase staining, which indicated that WNV infected neurons in the ventrolateral medulla (Figure 8B).

## Discussion

West Nile virus could conceivably cause death by a variety of mechanisms. Generalized neuropathy from widespread infection of the brain could occur and cause death by seizures with organ failure [30], but seizures are not common with human WNV infection [31] and pronounced seizures do not occur in rodent infections unless the virus is administered intranasally [32]. Moreover, focal neuropathy, not generalized neuropathy, probably accounts for the variability in disease signs and symptoms observed between individual human patients [18], [33], [34]. In the hamster model [2], [3], [17], disease signs occur independent of each other, i.e., they do not occur in a sequential pattern of disease progression. Therefore, this variability between individuals is likely due to the development of lesions in variable anatomical locations, and not simply due to WNV infection of the brain to cause generalized neuropathy. The discovery of the specific cause of death or the location of lesions responsible for other disease phenotypes is important to better manage the care of patients and to discover therapeutic interventions.

Other viral encephalitides, such as eastern equine encephalitis, causes cerebral vasculitis with associated microhemorrhages and edema [35]. In WNV-infected hamsters, cerebral vasculitis or hemorrhages were not histologically observed (data not shown). Cerebral edema was also not observed as indicated by measuring the wet and dry weights of brains, or by measuring permeability of Evans blue dye; therefore WNV-infected hamsters do not die from edema or hemorrhaging.

Experiments were performed to show that WNV-infected hamsters were not dying from dehydration or starvation, but that they were probably dying from neurological disease. For animal model development in which mortality is used as an end-point of the disease being investigated, one should know that death is not due simply to a loss of appetite and the resulting dehydration or starvation. To eliminate these possibilities, the effect of hydration on survival was determined, and blood chemistries were measured between moribund and not-moribund infected hamsters. The results showed that dehydration was not the cause of death, because hydration did not improve survival, weight change, or disease signs.

To interpret the results of blood chemistry analysis, one must have an understanding of the physiology of starvation. Glucose is the main sources of metabolic energy. In the absence of dietary sources, glucose is first obtained from breakdown of glycogen in the liver and muscle. After a short time when the available glycogen is expended, glucose is obtained from fats, which are metabolized to glycerol and free fatty acids [36]. The glycerol is used as a substrate to synthesize glucose. Fatty acids are also used directly as an energy source, but in the case of the brain where it is unavailable, the liver produces ketone bodies from fatty acid metabolism that can cross the blood-brain-barrier to supply the brain with energy. With advanced fasting, tissue proteins begin to be metabolized to produce amino acids, which can be converted to glucose in the liver, and become available to keep the brain functioning [37]. Finally, the loss of tissue protein affects vital organ functions to ultimately result in death, which in general is cardiac arrest from electrolyte imbalance and tissue damage.

The overall profile of the clinical tests indicated that the moribund animals were in a state of anorexia, but not starvation. Statistically higher levels of ketones, alkaline phosphatase (ALP), and blood urea nitrogen (BUN) in the moribund-infected animals as compared to the sham-infected animals indicated that protein catabolism was occurring, but high levels of glucose in both moribund and not-moribund infected animals indicated that energy reserves were still available. Plasma glucose in advanced stages of starvation would typically be very low; however, moribund and not-moribund infected hamsters had increased glucose compared to sham-infected hamsters. These elevated levels likely indicated that the glucose was being released from glycogen stores or synthesized from fatty acid metabolism, but did not indicate advanced starvation [38]. Ketosis of moribund-infected hamsters indicated that these animals were in a fasting mode, but not necessarily dying from starvation. Since creatine kinase is a marker for tissue damage, especially for that of heart or skeletal muscle, normal concentrations of creatine kinase suggest that the heart or other tissues were not being damaged from protein catabolism as seen in advanced starvation. Some protein catabolism may have been occurring, because blood urea nitrogen levels were elevated in some of the moribund infected hamsters. Both the dehydration and plasma chemistry data indicated that moribund hamsters were eating less than the not-moribund or sham-infected animals due to the WNND; but starvation were not the cause of death.

Obviously the death process involves the failure of vital organ functions such as cardiac and respiratory functions, but the cause of death will likely be a compilation of physiological events leading up to death. A range of abnormal ECGs were observed in WNV-infected hamsters, but only typically within hours or a day before mortality. Conversely, respiratory insufficiency was observed as early as 4 days before mortality. Moreover, the ECGs abnormalities were variable between dying hamsters, which did not suggest a common disease process leading to abnormal ECGs. However, respiratory patterns of plethysmography were similar, and all moribund mice had suppressed plethysmography results. Respiratory distress strongly correlated with mortality, unlike ECGs, motor unit number estimations [13], brain auditory evoked response [17], blood brain barrier permeability [28], and memory impairment [21]. Additionally, disease signs in hamsters including nose bleeding, front limb tremors, diarrhea, paralysis, and eye lacrimation were not correlated with mortality [2], [24]. Weight loss does correlate with mortality in rodents, but as demonstrated in this study, it was not the cause of death. Overall, respiratory insufficiency appears to be a good candidate for the cause of death in WNV-infected rodents.

Unrestrained whole body plethysmography is not as accurate in defining respiratory function as double-chamber plethysmography of restrained conscious animals for measuring airway resistance or conductance, or as a pulmonary function chamber where spirometry studies can be performed. For example, enhanced pause (Penh) cannot be used as a surrogate for airway resistance using whole body plethysmography [39]. Nevertheless, the composite of data presented in Figure 5 does provide some insight into the cause of respiratory insufficiency. For example, if the cause were lung disease such as with bronchioles constriction, peak expiratory flow would likely decline along with a compensatory increase in minute volume or tidal volume. Any lung disease resulting in increased CO_2_ would result in compensating respiratory functions. Yet, in the mouse plethysmography data, there were no signs of such compensating respiratory functions, i.e., the minute volume, tidal volume declined despite apparent lower peak expiratory or inspiratory flows, or frequency. Overall, the data are consistent with a neurological cause where the respiratory function is broadly declining.

One mouse (#206) that succumbed to the disease early after viral challenge on day 6 (Figure 5) did not appear to have the same level of severe plethysmography readings as animals that died later in the course of infection, although the data were trending toward abnormal values. This lower level of severity may have been due to the variability of the plethysmography assay; however, it may reflect a different disease process for occasional animals that succumb to the disease early as opposed to later. This possibility of different causes of death awaits further investigation.

To determine if extraneurological histopathology could account for respiratory insufficiency, the hearts, lungs, and diaphragms from 12 WNV-infected C57BL/6 mice were collected for H&E examination when minute volumes were clearly below two standard deviations of the mean. Tissues from 3 sham-infected mice were also collected. A board certified veterinary pathologist (see Acknowledgements) determined that there was no observable lung, heart, or diaphragm histopathology when comparing the WNV- with the sham-infected tissues (data not shown). As has been observed in hamsters, only one heart out of twelve WNV-infected mice had necrosuppurative vasculitis in the right atrium. Based on these data, extraneurological histopathology did not account for respiratory insufficiency or alterations of plethymographic readings.

The animal data of this study, and a poor prognosis for persons with respiratory insufficiency [8] support the hypothesis that neurological lesions affecting respiratory function is a cause of WNV-induced death, which is ultimately important in the management and treatment of West Nile neurological disease (WNND).

## Materials and Methods

### Ethics statement

This study was conducted in accordance with the approval of the Institutional Animal Care and Use Committee of Utah State University (IACUC approval #1079 and #1488; WNV APHIS Permit # 47210). The work was done in the AAALAC-accredited Laboratory Animal Research Center of Utah State University and in accordance with the National Institutes of Health Guide for the Care and Use of Laboratory Animals.

### Animals and viruses

Adult female Syrian golden hamsters, BALB/C mice, or C57BL/6 mice (>7 weeks old) were used (Charles River Laboratories). Animals were randomized to treatment groups by blindly selecting animals from a common container. WNV was diluted in minimal essential medium (MEM) immediately prior to subcutaneous (s.c.) injection in the groin area [17], [23], [28]. Hamsters were injected with a volume of 0.1 mL containing 5.7×10^7^ pfu of a New York WNV isolate from crow brain [40], [41]. Mice were injected with a volume of 0.1 mL containing 2.5×10^6^ pfu of a WN02 isolate designated as Kern 515 from Dr. Robert Tesh (Mosquito, 10/05/07, Kern County, CA, TVP 10799 BBRC lot # WNVKERN515-01, University of Texas Medical Branch Arbovirus Reference Collection). These two strains of WNV were used because of suitable mortality in the two rodent strains used and to demonstrate that the findings of this report are not rodent strain specific. Rodents were judged as moribund if they did not step forward if prodded, or if they did not right themselves when placed on their backs. Other disease signs, such as paralysis, front limb tremors, diarrhea, nose bleeding, eye lacrimation, or ruffled fur were not used as criteria for morbidity, because they do not correlate with mortality [2] (unpublished data). The only predictive sign of mortality is if the rodents cannot right themselves or if they will not step while being prodded.

### Plethysmography

Unrestrained whole body plethysmography was performed using a commercial plethysmograph with accompanying software (emka Technologies, Falls Church, VA). The mouse- and rat-sized plethysmograph chambers were manufactured by Buxco Research Systems with emka Technologies' catalogue numbers PLY 3211 and PLY 3213, respectively, which allow constant air to be pumped into and out of the chambers with equal pressure. The differential pressure due to breathing of the animal was acquired by acquisition and analysis software (IOX Base 2c, IOX 1 PULMO 2c, emka), and acquisition hardware (USB ACQ 4, emka). Amplifiers, digital transducers for measurements of temperature and humidity, and sensitive pressure transducers were also supplied (emka). Plethysmographs were calibrated before each day's use. Data were plotted using Prism 5 (GraphPad Sofware, Inc.). Eighteen parameters (minute volume, tidal volume, enhanced pause, end expiratory pause, end inspiratory pause, peak expiratory flow, peak inspiratory flow, frequency, inspiratory time expiratory time, relaxation time, pause, time delay, specific airway resistance, specific airway conductance, mid-expiratory flow) were calculated from the plethysmograph tracings, of which eight of the more interesting are displayed in Figure 5.

### Telemetry electrocardiography

Based on previous procedures [25], [42], [43], a midline dorsal incision was made in hamsters along the spine and a subcutaneous pocket was made to house the telemetric device (TA10EEAT-F20, Data Science International). Two recording-leads were subcutaneously tunneled toward the left and right clavicular region where the tips of leads were sutured to the pectoral muscles. The DSI ETA-F20 transmitter transmits temperature, ECG, and animal activity as an analog radio signal, with a digital signature. The duration of each reading was 2 min for every hour during the course of the experiment. The receivers, under the cages, were connected to a data acquisition matrix hard-wired to a PC-based computer running Dataquest A.R.T. Silver System software. During the course of the day, the resting or active states of hamsters were observed and recorded so that only data from resting hamsters were obtained. The data were acquired by acquisition and analysis software (IOX Base 2c, ECG Auto full, emka) with the use of software (ECG Auto DSI 4+, emka) to convert Dataquest DSI data for use with the emka software. To obtain only high quality ECG tracings, poor tracings were eliminated when the signal strength was low. Charts were made using Prism 5 (GraphPad Software, Inc.).

### Pulse oximetry

Saturated arterial oxygen (SaO_2_) measurements [27] were obtained from a mouse collar clip placed on the back of neck using the MouseOx™ pulse oximeter (STARR Life Sciences, Oakmont, PA) designed specifically to measure SaO_2_ levels in mice. Data were collected for a minimum 2–3 minutes per measurement (∼1000 data points) in a Windaq® waveform wave form file and converted to Microsoft® Excel. The averages were calculated.

### WNV immunofluorescent staining

Hamsters were anesthetized with ketamine/xylazine and then perfused directly with PBS and 4% paraformaldehyde after cardiac puncture. Paraffin sections (5 µm) were deparaffinized, rehydrated, antigen retrieved with DakoCytomation target retrieval solution in a pressure cooker for 15 min, and incubated with the 7H2 primary antibody and anti-neuron specific enolase (NSE) antibody (Chemicon,Temecula, CA) [23], [24]. Slides were examined using a Zeiss Axio observer equipped with a Vivatome module and images were captured using Nuance multispectral imaging 3.0.1.2 (CRi, Caliper Life Sciences, MA).

### Indicators of brain edema

The ratio of weights of brains before and after drying at 110°C for 24 hr was used as an indication of edema. As another indicator, blood-brain-permeability was determined by permeability of Evans blue dye penetrating into the brain [44]. Fifteen hamsters were injected with WNV and five were injected with sham. When one WNV-injected hamster became moribund, a corresponding non-moribund WNV-injected hamster was selected for jugular vein injection of Evans blue dye with 0.4 mL of 18 mg/mL in saline per 110 g hamster. Four hour later, the animals were necropsied and brains were examined for any blue color due to breakdown of the blood-brain-barrier.

### Hydration

Hamsters were treated s.c. with D5 dextrose solution (5% dextrose in 0.9% physiological saline) at a dosage of 100 mL/kg once per day beginning on day 6 after viral challenge until day 14. In the same way, control animals were sham-injected without any addition of solution. The animals were monitored through day 21 for A) mortality, B) weight change, and C) disease signs such as hind limb paralysis, front limb tremors, eye lacrimation, and diarrhea. Mortality was considered a disease sign when constructing the Figure 2C.

### Plasma assays

Plasma was collected using lithium-heparin and processed for blood chemistries, ketone, and creatine kinase. Chemistry analyzer (VetScan, Abaxis, CA) designed to measure alanine aminotransferase, alkaline phosphatase, albumin, total bilirubin, creatine, blood urea nitrogen, sodium, potassium, calcium, phosphorous, amylase, glucose, total protein, and globulin was used. Kits were used to measure ketones (beta-hydroxybutyrate) (Cayman Chemical Co., Ann Arbor, MI) and creatine kinase (Enzychorm creatine kinase assay kit, Bioassay Systems, Hayward, CA).

### Statistics

Survival data were analyzed by log rank test. Plasma ketone, creatine kinase and blood chemistry data were analyzed by the t-test. Pulse oximetry data were analyzed by one-way analysis of variance with Newman-Keuls comparison. For analysis of mouse plethysmography data, two standard deviations of the mean at days −2, 1, and 2 for all animals were calculated and represented as dotted lines in Figure 5. For analysis of hamster plethysmography data in Figure 7, the dotted line represents two standard deviations of sham-injected hamsters. Data within this range were considered normal, and those outside were considered abnormal.

## References

[1] Lindsey NP, Staples JE, Lehman JA, Fischer M (2010). Surveillance for human West Nile virus disease - United States, 1999–2008.. MMWR Surveill Summ.

[2] Morrey JD, Day CW, Julander JG, Olsen AL, Sidwell RW (2004). Modeling hamsters for evaluating West Nile virus therapies.. Antiviral Res.

[3] Xiao SY, Guzman H, Zhang H, Travassos da Rosa AP, Tesh RB (2001). West Nile virus infection in the golden hamster (Mesocricetus auratus): a model for West Nile encephalitis.. Emerg Infect Dis.

[4] Oliphant T, Engle M, Nybakken GE, Doane C, Johnson S (2005). Development of a humanized monoclonal antibody with therapeutic potential against West Nile virus.. Nat Med.

[5] Leis AA, Stokic DS (2005). Neuromuscular Manifestations of Human West Nile Virus Infection.. Curr Treat Options Neurol.

[6] Saad M, Youssef S, Kirschke D, Shubair M, Haddadin D (2005). Acute flaccid paralysis: the spectrum of a newly recognized complication of West Nile virus infection.. J Infect.

[7] Shpall AI, Varpetian A, Ginsberg DA (2003). Urinary retention in a patient with West Nile virus.. Urology.

[8] Sejvar JJ, Bode AV, Marfin AA, Campbell GL, Pape J (2006). West Nile Virus-associated flaccid paralysis outcome.. Emerg Infect Dis.

[9] Sejvar JJ (2007). The long-term outcomes of human West Nile virus infection.. Clin Infect Dis.

[10] Sejvar JJ, Curns AT, Welburg L, Jones JF, Lundgren LM (2008). Neurocognitive and functional outcomes in persons recovering from West Nile virus illness.. J Neuropsychol.

[11] Carson PJ, Konewko P, Wold KS, Mariani P, Goli S (2006). Long-term clinical and neuropsychological outcomes of West Nile virus infection.. Clin Infect Dis.

[12] Morrey JD, Siddharthan V, Wang H, Hall JO, Skirpstunas RT (2008). West Nile virus-induced acute flaccid paralysis is prevented by monoclonal antibody treatment when administered after infection of spinal cord neurons.. J Neurovirol.

[13] Siddharthan V, Wang H, Motter NE, Hall JO, Skinner RD (2009). Persistent West Nile virus associated with a neurological sequela in hamsters identified by motor unit number estimation.. J Virol.

[14] Wang H, Siddharthan V, Hall JO, Morrey JD (2009). West Nile virus preferentially transports along motor neuron axons after sciatic nerve injection of hamsters.. J Neurovirol.

[15] Fratkin JD, Leis AA, Stokic DS, Slavinski SA, Geiss RW (2004). Spinal cord neuropathology in human West Nile virus infection.. Arch Pathol Lab Med.

[16] Hunsperger EA, Roehrig JT (2006). Temporal analyses of the neuropathogenesis of a West Nile virus infection in mice.. Journal of Neurovirology.

[17] Morrey JD, Siddharthan V, Wang H, Hall JO, Motter NE (2010). Neurological suppression of diaphragm electromyographs in hamsters infected with West Nile virus.. J Neurovirol.

[18] Sampson BA, Ambrosi C, Charlot A, Reiber K, Veress JF (2000). The pathology of human West Nile Virus infection.. Hum Pathol.

[19] Agamanolis DP, Leslie MJ, Caveny EA, Guarner J, Shieh WJ (2003). Neuropathological findings in West Nile virus encephalitis: a case report.. Ann Neurol.

[20] Doron SI, Dashe JF, Adelman LS, Brown WF, Werner BG (2003). Histopathologically proven poliomyelitis with quadriplegia and loss of brainstem function due to West Nile virus infection.. Clin Infect Dis.

[21] Smeraski CA, Siddharthan V, Morrey JD (2011). Treatment of spatial memory impairment in hamsters infected with West Nile virus using a humanized monoclonal antibody MGAWN1.. Antiviral Research.

[22] Smee DF, Hurst BL, Wong MH, Tarbet EB, Babu YS (2010). Combinations of oseltamivir and peramivir for the treatment of influenza A (H1N1) virus infections in cell culture and in mice.. Antiviral Res.

[23] Morrey JD, Siddharthan V, Olsen AL, Roper GY, Wang HC (2006). Humanized monoclonal antibody against West Nile virus E protein administered after neuronal infection protects against lethal encephalitis in hamsters.. Journal of Infectious Disease.

[24] Morrey JD, Siddharthan V, Olsen AL, Wang H, Julander JG (2007). Defining limits of treatment with humanized neutralizing monoclonal antibody for West Nile virus neurological infection in a hamster model.. Antimicrob Agents Chemother.

[25] Wang H, Siddharthan V, Hall JO, Morrey JD (2011). Autonomic nervous dysfunction in hamsters infected with West Nile virus.. PLoS ONE.

[26] Groeben H, Meier S, Tankersley CG, Mitzner W, Brown RH (2003). Heritable differences in respiratory drive and breathing pattern in mice during anaesthesia and emergence.. British journal of anaesthesia.

[27] Sidwell RW, Huffman JH, Gilbert J, Moscon B, Pedersen G (1992). Utilization of pulse oximetry for the study of the inhibitory effects of antiviral agents on influenza virus in mice.. Antimicrob Agents Chemother.

[28] Morrey JD, Olsen AL, Siddharthan V, Motter NE, Wang H (2008). Increased blood-brain barrier permeability is not a primary determinant for lethality of West Nile virus infection in rodents.. J Gen Virol.

[29] Mandel DA, Schreihofer AM (2006). Central respiratory modulation of barosensitive neurones in rat caudal ventrolateral medulla.. The Journal of physiology.

[30] Gardner CL, Ebel GD, Ryman KD, Klimstra WB (2011). Heparan sulfate binding by natural eastern equine encephalitis viruses promotes neurovirulence.. Proceedings of the National Academy of Sciences of the United States of America.

[31] Bagic A, Boudreau EA, Greenfield J, Sato S (2007). Electro-clinical evolution of refractory non-convulsive status epilepticus caused by West Nile virus encephalitis.. Epileptic disorders : international epilepsy journal with videotape.

[32] Getts DR, Matsumoto I, Muller M, Getts MT, Radford J (2007). Role of IFN-gamma in an experimental murine model of West Nile virus-induced seizures.. J Neurochem.

[33] Weiss D, Carr D, Kellachan J, Tan C, Phillips M (2001). Clinical findings of West Nile virus infection in hospitalized patients, New York and New Jersey, 2000.. Emerg Infect Dis.

[34] Bode AV, Sejvar JJ, Pape WJ, Campbell GL, Marfin AA (2006). West Nile virus disease: a descriptive study of 228 patients hospitalized in a 4-county region of Colorado in 2003.. Clin Infect Dis.

[35] Paessler S, Aguilar P, Anishchenko M, Wang HQ, Aronson J (2004). The hamster as an animal model for eastern equine encephalitis–and its use in studies of virus entrance into the brain.. The Journal of infectious diseases.

[36] Berdanier CD (2000). Advanced nutrition: Macronutrients; 2nd, editor.. Boca Raton: CRC Press.

[37] Yudkoff M, Daikhin Y, Nissim I, Lazarow A (2001). Brain amino acid metabolism and ketosis.. Journal of neuroscience research.

[38] Hoffer LJ, Shils ME (2006). Metabolic consequences of starvation..

[39] Bates J, Irvin C, Brusasco V, Drazen J, Fredberg J (2004). The use and misuse of Penh in animal models of lung disease.. American journal of respiratory cell and molecular biology.

[40] Lanciotti RS, Ebel GD, Deubel V, Kerst AJ, Murri S (2002). Complete genome sequences and phylogenetic analysis of West Nile virus strains isolated from the United States, Europe, and the Middle East.. Virology.

[41] Lanciotti RS, Kerst AJ (2001). Nucleic acid sequence-based amplification assays for rapid detection of West Nile and St. Louis encephalitis viruses.. J Clin Microbiol.

[42] Sgoifo A, Stilli D, Medici D, Gallo P, Aimi B (1996). Electrode positioning for reliable telemetry ECG recordings during social stress in unrestrained rats.. Physiol Behav.

[43] Mongue-Din H, Salmon A, Fiszman MY, Fromes Y (2009). Periodic variation in R-R intervals and cardiovascular autonomic regulation in young adult Syrian hamsters.. Am J Physiol Regul Integr Comp Physiol.

[44] Gowen BB, Julander JG, London NR, Wong MH, Larson D (2010). Assessing changes in vascular permeability in a hamster model of viral hemorrhagic fever.. Virol J.

